# 3D C-arm navigated suture button implantation for AC joint dislocations - the pilot study

**DOI:** 10.1007/s00068-024-02582-z

**Published:** 2024-07-12

**Authors:** Alexander Böhringer, Florian Gebhard, Christoph Dehner, Alexander Eickhoff, Raffael Cintean, Carlos Pankratz, Konrad Schütze

**Affiliations:** Department of Trauma Hand and Reconstructive Surgery Ulm University, Albert-Einstein-Allee 23, Ulm, 89081 Germany

**Keywords:** 3D C-arm imaging, Computer navigation, Shoulder TightRope, AC joint, Tossy, Rockwood

## Abstract

**Purpose:**

The surgical treatment of acute traumatic AC joint dislocations is still a subject of scientific debate in the literature. The arthroscopically assisted stabilization procedure with a suture button system has been successfully established and is widely used in daily practice. It is minimally invasive and allows the anatomical reconstruction of the torn coracoclavicular ligaments in one step with a permanent implant that does not have to be removed in a second operation. This clinical pilot study is the first to describe the new method of navigated suture button implantation with the future aim of further reducing surgical invasiveness and further increasing surgical precision.

**Materials and methods:**

10 patients with a Rockwood 3b/5 injury could be included in the prospective study (DRKS00031855) within 5 months according to inclusion and exclusion criteria. Surgical stabilization was performed with a suture button system via a navigated coracoclavicular drill tunnel. Demographic and radiological data as well as information on health and shoulder function were collected from patient records, X-rays, DVT scan and 3 questionnaires (DASH, NHS and Eq. 5D) at the preoperative, intraoperative and postoperative (discharge, 6 weeks and 3 months) time points.

**Results:**

All operations could be performed within 8.8 days (± 6.81) after trauma. The average operation time was 50.3 min (± 8.81). The mean distance of the drill hole in the clavicle to the AC joint was 26.6 mm (± 2.63). The radiologically measured vertical coracoclavicular distance was 38.8 mm (± 6.16) at discharge and 41.11 mm (± 7.51) at 3 months. This loss of reduction was not statistically significant. In contrast, the DASH, NHS and Eq. 5D results showed significant improvement from discharge to 3 months postoperatively.

**Conclusion:**

Image-guided 3D C-arm navigated AC joint suture button stabilization is feasible in everyday surgical practice. It may be possible to achieve a further reduction in invasiveness while at the same time increasing the accuracy of implant positioning. Further clinical studies with a larger number of patients and a longer follow-up period are necessary to enable a comparison with conventional methods.

**Supplementary Information:**

The online version contains supplementary material available at 10.1007/s00068-024-02582-z.

## Introduction

AC (acromioclavicular) joint dislocations were first described in detail by Tossy et al. in 1963 and classified into three grades of vertical instability [1]. Rockwood et al. expanded the classification in 1984 to include three additional degrees of severity, which also took into account a fixed dislocation to the dorsal side [2]. In a consensus statement of the ISAKOS (International Society of Arthroscopy, Knee Surgery & Orthopaedic Sports Medicine) Upper Extremity Committee in 2014, the third degree of severity (tearing of all ligaments and dislocation of the lateral clavicle end by shaft width to the cranial) was divided into a (horizontally stable) and b (horizontally unstable), depending on additional horizontal instability, because there is no clear treatment recommendation for or against surgical stabilization in the literature here to date [3].

Currently, the indication for surgical stabilization in the European region is generally given from Rockwood 3b to 6, with severity grades 4 and 6 being extremely rare [3, 4]. Both the hook plate and the arthroscopic suture button procedure are currently used in everyday practice. The hook plate is used more in traumatology [5], the suture button procedure more in orthopedics and specialized centers [6].

Advantages of the arthroscopic suture button method include significantly better clinical outcomes, significantly better return to sport and return to athletic level, lower rate of horizontal instability, higher patient satisfaction and quality of life, no need for secondary removal of osteosynthesis material, a lower revision rate, and no risk of direct rotator cuff damage or osteolysis/fracture of the acromion [7–10].

General disadvantages and complications of the arthroscopic suture button method are described by Woodmass et al. in a systematic review of the literature [11]. Loss of reduction (26.8%), implant-related problems (25%), fractures of clavicle or coracoid (5.3%), and infections (3.8%) are mentioned. Not mentioned are specific shoulder motion restrictions after general shoulder arthroscopy and tunnel widening due to implant bone contact during mobilization. In addition, several prognostic factors for an inferior postoperative outcome have been reported. These include older patient age, high BMI, nicotine abuse, late timing of surgery, and inadequate AC joint reduction as well as inaccurate implant position [12]. Therefore, a good patient selection, time of surgery, and increasingly minimally invasive methods are being sought [13].

Due to these remaining problems, there is still a need for further research. To improve the intraoperative visual assessment of joint position and bony configuration as well as the accuracy of drilling and implant positioning while simultaneously reducing invasiveness, image-guided computer-navigated drill tunnel placement could be an option. The requirement of intraoperative DVT (digital volume tomography) imaging at the shoulder has already been demonstrated [14]. Even though only cement-augmented plate osteosynthesis for proximal humerus fractures was examined, the neighboring AC joint can also be imaged multiplanar. This creates the prerequisite for image-guided computer navigation of the shoulder. However, this has so far only been investigated in vitro on cadaver shoulders and synthetic shoulder models [15, 16].

It is anticipated that the precise intraoperative 3D visualization may enable surgeons to achieve the most anatomically AC joint position possible. It is also anticipated that creating a navigated coracoclavicular drill tunnel could enable surgeons to position the implant as precisely as possible, while at the same time further reducing invasiveness.

The aim of this study was on the one hand to demonstrate the feasibility of the method clinically, and on the other hand to find out the short-term outcome of the treated patients on the basis of radiological measurement data and questionnaires on complaints and function of the affected shoulder. It was not the aim to compare the new method directly with the established and common methods of the hook plate and the arthroscopic/open suture button method. It was also not the aim of this study to compare the navigated single button system with a double suture button system or an additive horizontal cerclage.

## Materials and methods

Approval was first obtained from the local ethics committee for this prospective clinical feasibility study (application number 88/23). From April to August of the year 2023, 10 patients were included in the study that was registered under the number DRKS00031855 in the DRKS (Deutsches Register Klinischer Studien - german register of clinical studies). Inclusion criteria were all patients 18 years of age and older with an acute AC joint (acromioclavicular joint) injury Rockwood 3b and 5 [2, 3] with a desire for surgical therapy and consent to participate in the study. Patients were included via consultation and emergency department of our level 1 trauma center in Germany and informed accordingly about the procedure and the study. Exclusion criteria were open injuries or infected wounds in the surgical site, fractures of the acromion, clavicle, or coracoid, and injuries older than 3 weeks, recurrent injuries, apparent incompliance and dementia, Rockwood 4 and 6 injuries, known prior AC joint surgery, existing pregnancy, and concomitant neurovascular injuries.

**Fig. 1:**
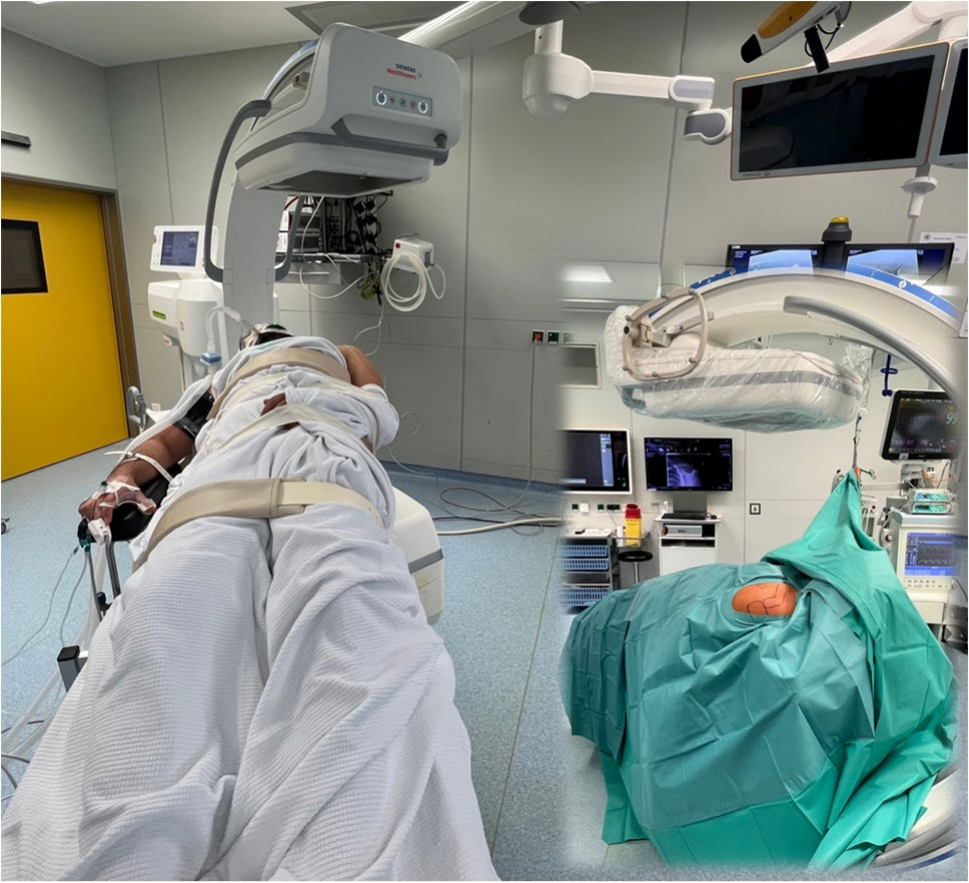
Patient positioning on a carbon table in a beach chair like position in the OR is shown. The mobile C-arm is positioned at the head end at a slight angle to the table. After disinfection and covering with sterile drapes, the affected AC joint / shoulder remains accessible as the operating area. The bony landmarks (clavicle, acromion and coracoid process) are marked

**Fig. 2:**
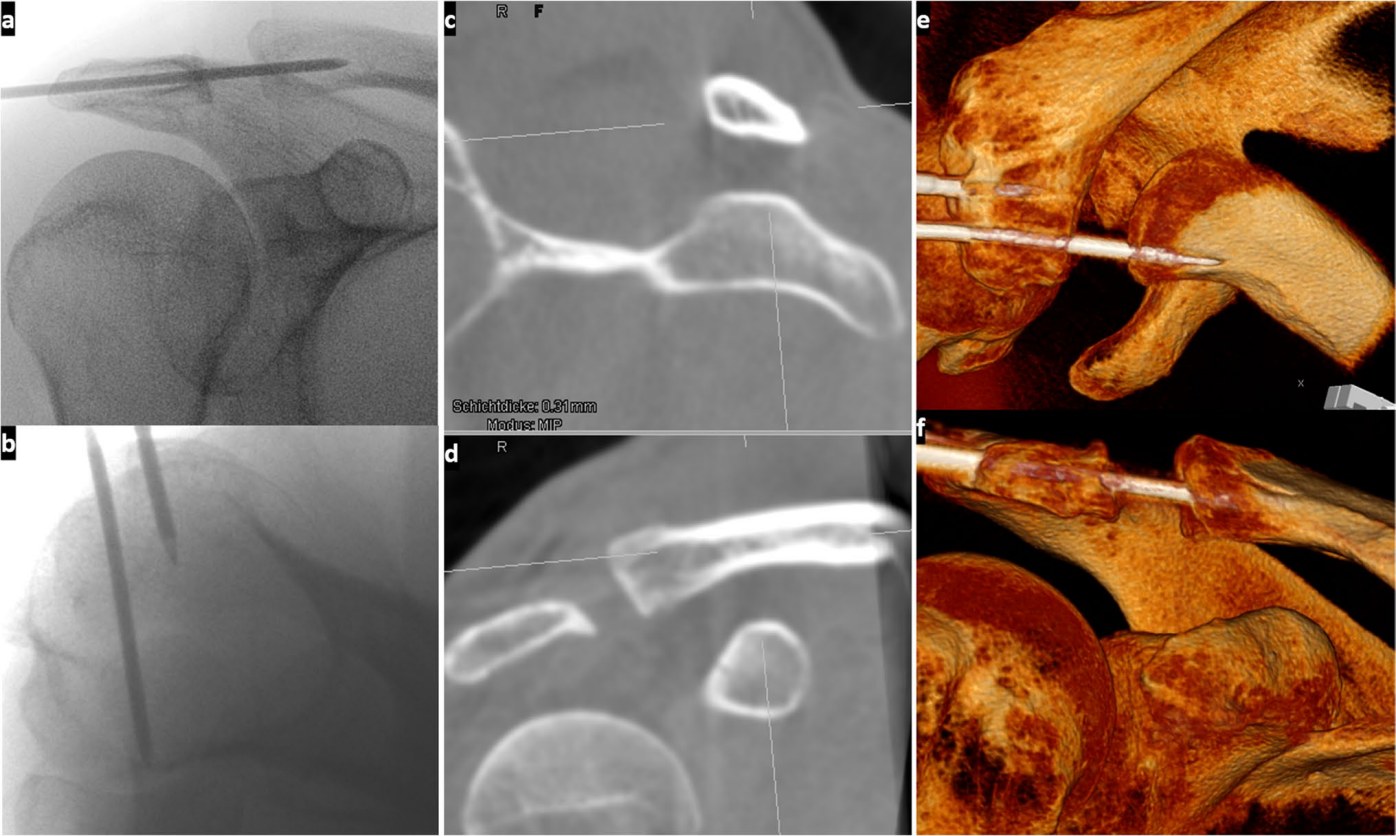
X-ray images after closed AC joint reduction and Kirschner wire retention are shown in a (coronal) and b (axial). The second wire is used to attach the first reference for computer navigation to the patient’s acromion. The second reference is located on the C-arm (see Fig. 1). In c and d parasagittal and coronal DVT slices can be seen, in e and f 3D reconstructions for exact assessment of the AC joint position

For surgery, patients were positioned on a carbon table in beach chair like position in the operating room with gonadal protection and lead goggles (see Fig. 1). After closed reduction of the AC joint, a Kirschner wire was inserted percutaneously through the lateral acromion edge for temporary transfixation. A Y-shaped reference device with three spheres was attached to the free end of the wire. A second reference device was attached to the mobile isocentric C-arm image intensifier (Siemens® Cios-Spin). The navigation camera was aimed at both references simultaneously at the start of the 3D scan. The DVT image data was then transferred to the navigation software (Brainlab “Buzz”, consisting of hardware (instruments, references, cameras and screens) and software (Backbone 1.6.2.54, Backbone Viewer 1.6.2.578, Brainlab Buzz 1.0.0.12 and Brainlab Nodemaster 1.6.0.48)) and the AC joint reduction could be precisely assessed (see Fig. 2).

**Fig. 3:**
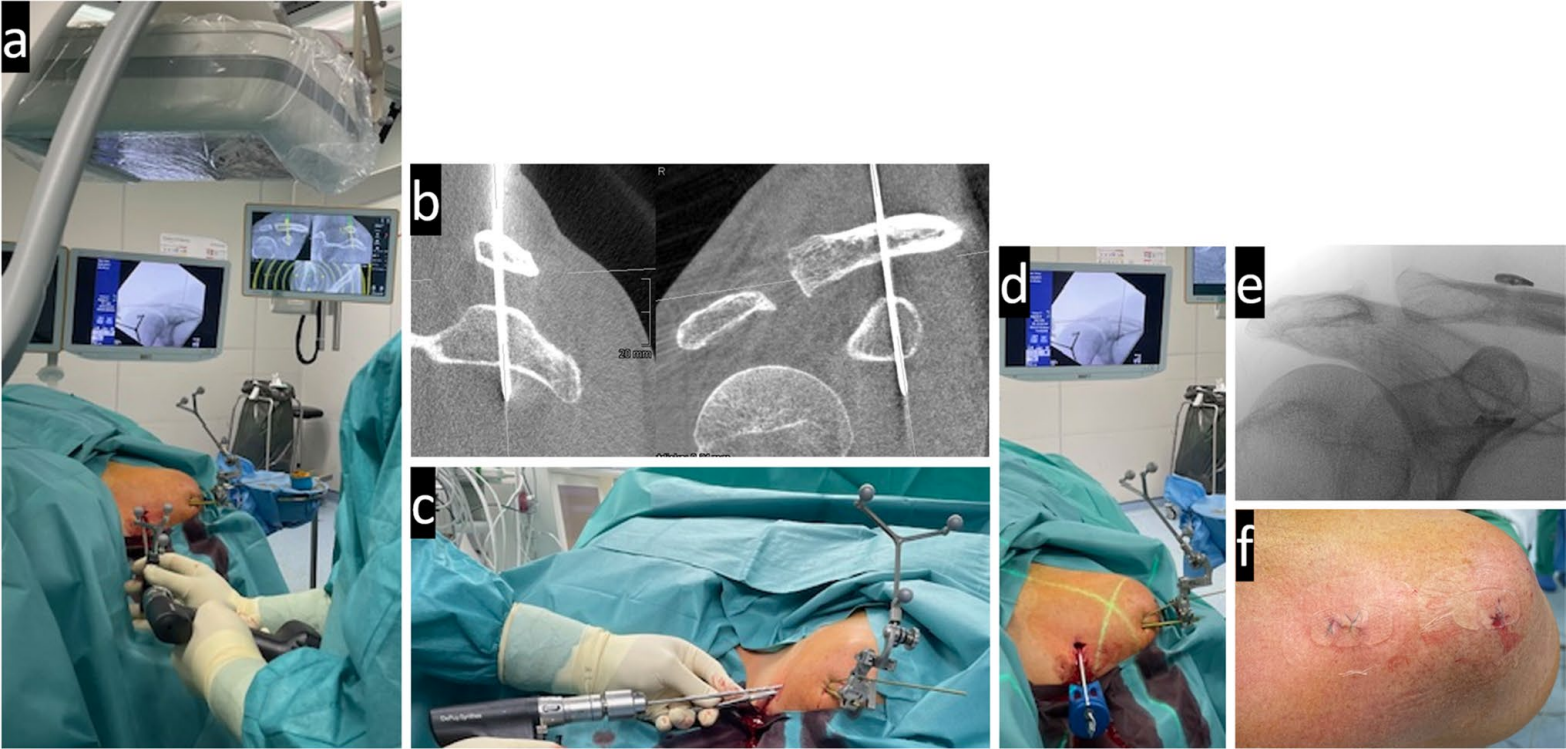
a shows the computer-navigated insertion of the 1.8 mm Kirschner wire according to the planning (shown in the upper right screen) under additional X-ray control (upper left monitor). In b, a parasagittal and a coronal slice of the second DVT can be seen for exact position control of the inserted wire. The 4 mm cannulated overdrilling of the wire is shown in c. Subsequently, d shows the insertion of the implant under X-ray control (monitor at the upper edge of the image). e and f show the radiological and clinical result with the implant tied in place and closed stitch incisions

After planning the CC (coracoclavicular) drill tunnel on the navigation monitor (the center of the drill hole was aimed at 25–30 mm to the lateral clavicular margin [17]), a 1.8 mm Kirschner wire was inserted using computer navigation. This was followed by drilling with a cannulated drill over the K-wire. The implant (Arthrex AC TightRope® Suture Button System) could then be inserted through this drill tunnel and tightened (see Fig. 3). A slight overcorrection was aimed for [13].

For rehabilitation, the shoulder was protected in a Gilchrist bandage for two weeks postoperatively until the stitches were removed. The patient was then allowed to move the shoulder freely below the horizontal without weight-bearing until the end of the sixth postoperative week. A clinical-radiological follow-up was then carried out to allow pain-adapted gradual movement and subsequent weight-bearing. 3 months postoperatively, a further clinical-radiological follow-up was carried out for complete release.

**Fig. 4:**
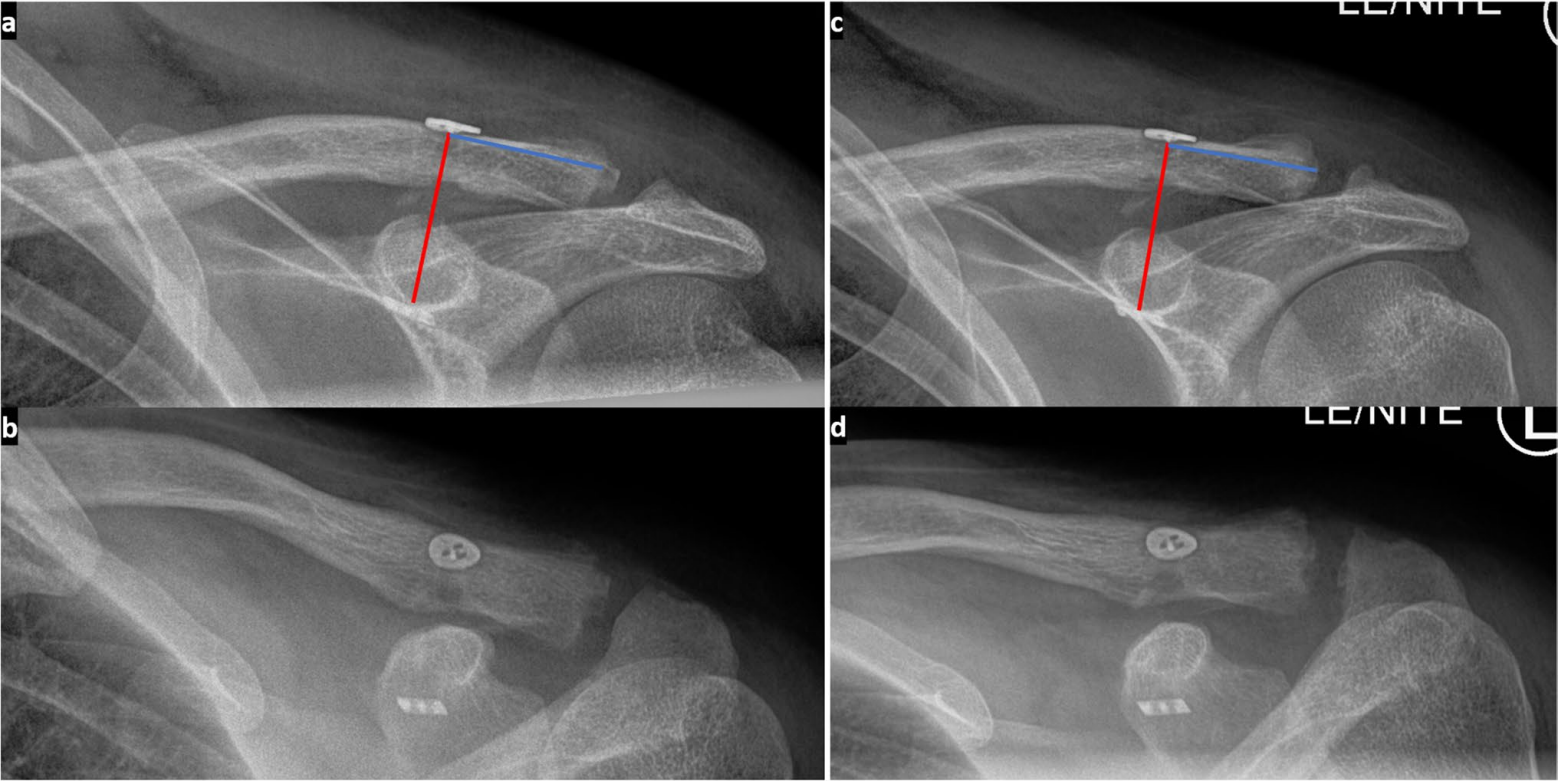
The collection of radiological measurement data is demonstrated here on two X-ray controls in two planes each. The anterior posterior (a.p. / coronal) AC joint images at discharge (a) and after 3 months (c) show the measured CC distance (vertical AC joint congruence) between the titanium plates in mm (red line in each case) and the measured distance from the center of the clavicle drill hole to the lateral end of the clavicle in mm (blue line in each case). In the second plane (b at discharge and d after 3 months in oblique / axial), an assessment of the horizontal AC joint congruence is possible

To determine AC joint reduction, drill channel position and implant position as well as CC and AC distance, radiological imaging (X-ray and DVT) was evaluated and compared preoperatively, intraoperatively, as well as in the postoperative course. The computer program DeepUnity® (Diagnost 1.2.0.1, Dedalus) was used for this purpose (see Fig. 4).

Three adapted questionnaires were used to determine complaints and function of the affected shoulder in daily life. The DASH (disabilities of the arm, shoulder and hand) [18], Eq. 5D (a self-assessed, health related, quality of life questionnaire) [19] and NHS (nottingham clavicle score) [20] test. Data collection took place in each patient at specified time points-preoperatively, postoperatively, at 6 weeks, and at 3 months (see Fig. 5 in the supplementary material).

Data analysis was performed with Microsoft Excel (2019 MSO) and IBM SPSS Statistics (V27.0). Demographic characteristics were described as means and as standard deviations, as well as medians and ranges. Due to the small numbers comparisons between the different investigation points were done by kruskal wallis test and post hoc bonferroni correction.

## Results

The targeted number of 10 patients for this pilot study was reached within 5 months. All patients were male and the average age was 47.8 ± 15.51 years. Nine patients could be fully followed up by the planned end of the study (3 months postoperatively), one patient withdrew from the follow-up.

All operations could take place within the targeted 3 weeks after the respective trauma. The average was 8.8 days with a standard deviation of ± 6.81.

The surgical procedure presented for the first time in this pilot study could be performed successfully in all cases. The average operating time was 50.3 ± 8.81 min. There were no significant intraoperative (such as fracture of the coracoid process/clavicle/acromion, implant malpositioning or damage to nerves, vessels and organs) or postoperative complications (such as infection, wound healing problems, significant post-operative bleeding, complete implant failure or secondary fractures).

The mean distance of the drill hole in the clavicle to the AC joint (drill hole center to lateral clavicle end) was 26.6 ± 2.63 mm).

The radiologically measured vertical CC distance between the two titanium plates above the clavicle and below the coracoid process was 38.8 ± 6.16 mm at discharge and 41.11 ± 7.51 mm after 3 months. This difference in vertical length increase was not statistically significant.

The results of the demographic and geometric variables are shown in Table 1 below.

**Table 1:** The mean values and standard deviations of six defined variables are listed here

variable	mean value	standard deviation
age	47.80	15.51
time to surgery in days	8.80	6.81
op time in minutes	50.30	8.81
drill hole to AC joint distance in mm	26.60	2.63
CC distance at discharge in mm	38.80	6.16
CC distance at 3 month in mm	41.11	7.51

The results of the evaluation of the questionnaires are shown in Table 2. The DASH, the NHS and the Eq. 5D showed a significant improvement in outcome between the assessment points at discharge and 3 months postoperatively. In detail, the DASH showed an improvement in score from 115.89 to 51.22 (worst possible score 150, best possible score 30), the NHS showed an improvement from 50.22 to 74.89 (worst possible score 20, best possible score 100), the Eq. 5D showed an improvement from 13.89 to 7.89 (worst possible score 25, best possible score 5) and the Eq. 5D VAS showed an improvement from 54.44 to 88.78 (worst possible score 0, best possible score 100)

**Table 2:** The table shows the results of the DASH (disabilities of the arm, shoulder and hand), NHS (nottingham clavicle score), Eq. 5D (health related quality of life questionnaire) and Eq. 5D VAS (health related quality of life questionnaire as a visual analog scale) questionnaires at the time of discharge, after 6 weeks and after 3 months, each as a mean value and standard deviation

Questionnaires	DASH	NHS	Equation 5D	Equation 5D VAS
values on discharge	115.89 ± 11.33	50.22 ± 5.14	13.89 ± 2.57	54.44 ± 19.44
values at 6 weeks	77.00 ± 19.35	63.22 ± 11.70	9.11 ± 1.27	78.11 ± 10.52
values at 3 month	51.22 ± 14.46	74.89 ± 12.25	7.89 ± 1.90	88.78 ± 10.91

**Fig. 5:**
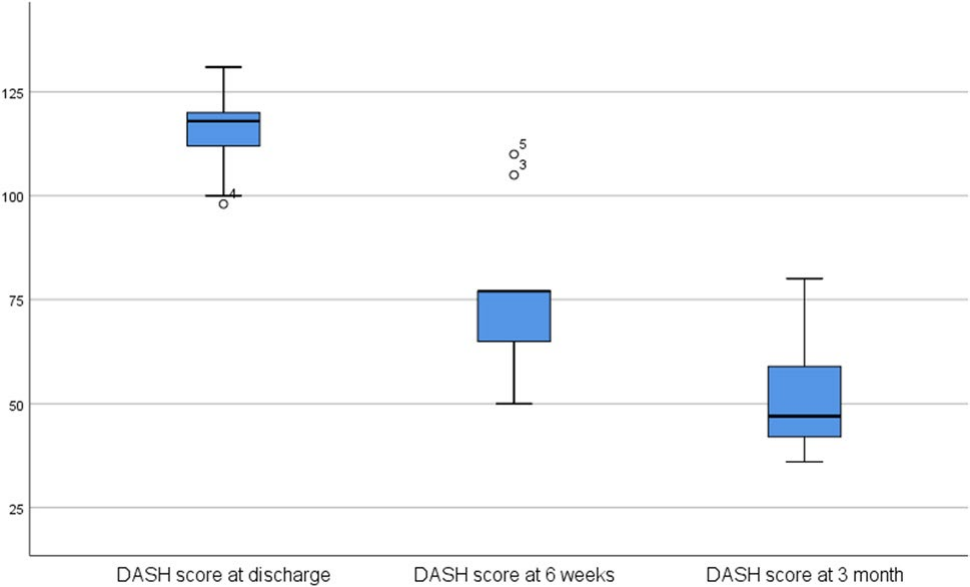
The boxplot diagram of the DASH results shows the scores on the y-axis and the three time points of the survey on the x-axis. The significance level is 0.05. The difference between all three time points is significant (*p* = 0.034)

**Fig. 6:**
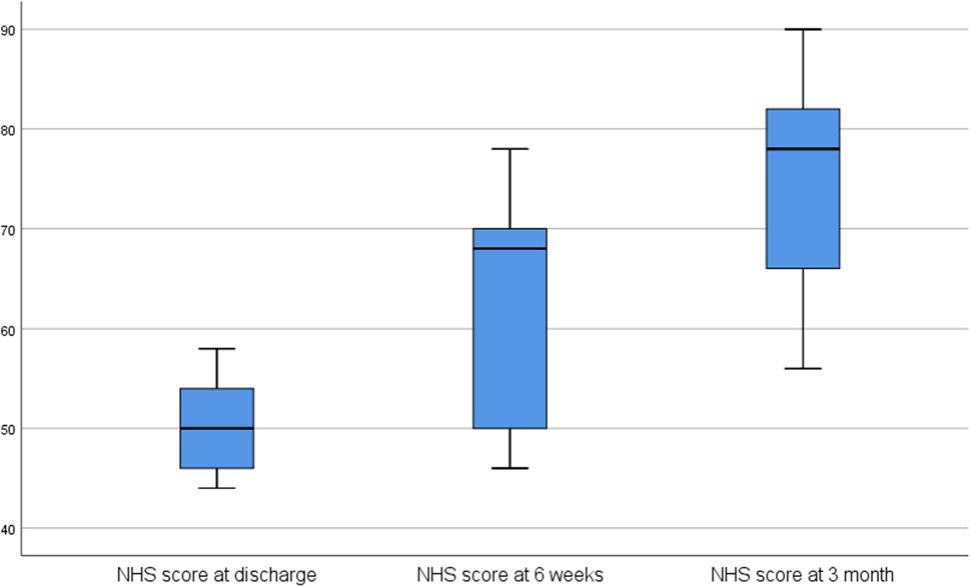
The boxplot diagram of the NHS results shows the scores on the y-axis and the three time points of the survey on the x-axis. The significance level is 0.05. The difference between the time points discharge − 3 months and 6 weeks − 3 months is significant (*p* = 0.045)

**Fig. 7:**
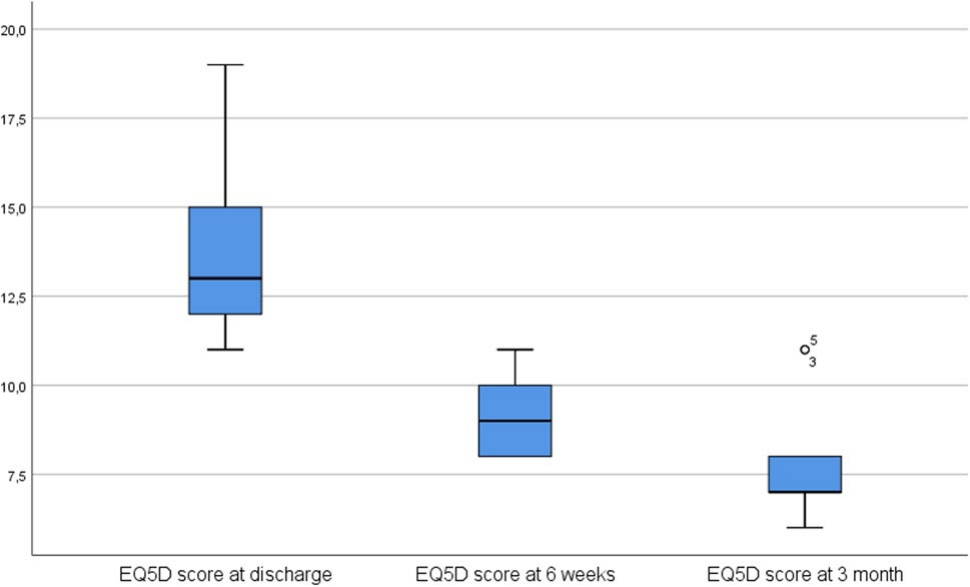
The boxplot diagram of the Eq. 5D results shows the scores on the y-axis and the three survey time points on the x-axis. The significance level is 0.05. The difference between the time points discharge − 6 weeks and discharge − 3 months is significant (*p* = 0.018)

**Fig. 8:**
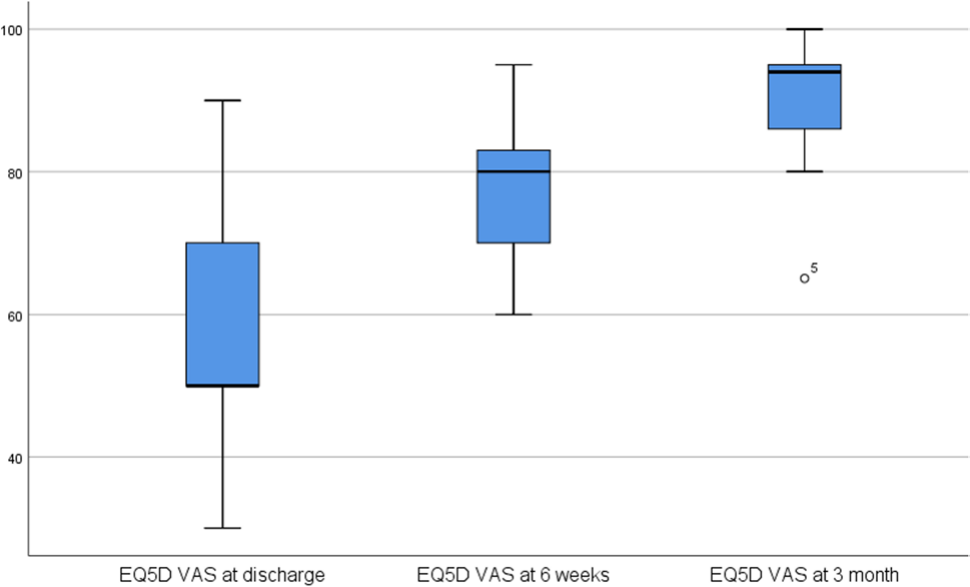
The boxplot diagram of the Eq. 5D VAS results shows the scores on the y-axis and the three time points of the survey on the x-axis. The significance level is 0.05. The difference between the time points discharge − 3 months and 6 weeks − 3 months is significant (*p* = 0.013)

The results of the four respective questionnaires are analyzed as boxplots below. In each case, the median value, the upper and lower quartiles, the minimum and maximum values and the outliers at the time of discharge, after 6 weeks and after 3 months are shown (Figs. 5, 6, 7 and 8).

## Discussion

The main aim of this study was to demonstrate the feasibility of the navigated suture button method clinically for the first time. In addition, short-term results on complaints and function of the operated shoulders were determined from this small patient group. The aim of this study was not to compare the new method directly with the established and common procedures of the hook plate and the arthroscopic / open suture button method. It was also not the aim of this study to compare the navigated single suture button system with a double suture button system or an additive horizontal cerclage.

The navigated method has so far only been investigated in vitro once on 10 synthetic shoulder models [15] and once on nine cadaveric shoulders [16]. However, in the studies by Stübig et al. [16, 21, 22] as well as in the studies by Theopold et al. [23–25] for intraoperative 3D imaging of the shoulder, a mobile C-arm image intensifier with a variable isocenter transthoracic from the opposite side, was used. In contrast, in the present study the navigated creation of the CC drill tunnel was simple, reliable and, with an average operating time of 50 min for the entire procedure, already well feasible under everyday clinical conditions with a common isocentric C-arm at the patient’s head end.

In accordance with the current literature for the arthroscopic suture button method, the navigated procedure also showed a quick and significant improvement in complaints and function of the operated shoulders in all 3 questionnaires (DASH, NHS, Eq. 5D + EQ5D VAS) between discharge and 3 months postoperatively [11, 26–31].

With confirmation of the clinical feasibility, further aims of the study were to investigate possible advantages of the image-guided navigated procedure in terms of accurate visual assessment, precise implant positioning and low invasiveness.Firstly, visualisation: An intraoperative 3D DVT is taken after closed AC joint reduction and temporary K-wire transfixation. This allows the joint position to be checked precisely and corrected immediately if necessary. A confirmed anatomical AC joint position at the time of unidirectional drill tunnel creation and implant positioning is certainly advantageous. Several studies in the literature have already shown that intraoperative 3D imaging, including shoulder imaging, is superior to conventional X-ray imaging in terms of the ability to assess fractures, joint positions and implant positions [15, 23, 24]. Furthermore, it is generally known that joint injuries that are restored as anatomically as possible lead to a better postoperative result [12].

Secondly, precision: The image-guided navigation for creating the CC drill tunnel should enable the most precise possible implantation of the suture button implant. For example Theopold et al. have already demonstrated a higher accuracy in CC drill tunnel placement by using navigation instead of an arthroscopic targeting device alone (with the targeting device alone, 30% of the first drillings were inaccurate and a corresponding revision was necessary. With the optoelectronic navigation system, all drillings were immediately accurate) [32]. In another study on 9 cadaveric shoulders, navigated transfixation of the AC joint was shown to be more accurate than 8 wires inserted freehand under x-ray control [21]. Further studies confirm the significantly higher accuracy of surgical procedures through the use of 3D imaging and navigation [15, 16, 23, 32].

Thirdly, invasiveness: Only a single stab incision above the clavicle is required for navigated drill tunnel creation and implant positioning. This makes the procedure as minimally invasive as possible. There is broad consensus in the literature on the importance of minimally invasive surgical methods [33–35]. As described for the open or arthroscopically assisted suture button procedure, no increased risks or complications such as infection, wound healing problems, bleeding, fracture and damage to nerves, vessels or organs have occurred with the new navigated method to date [7–11]. By further reducing invasiveness, less internal damage (e.g. injury to the rotator cuff, long biceps tendon, labrum and glenohumeral ligaments) and the absence of postoperative frozen shoulder, which is reported to be between 2.7 and 15%, may be expected [36, 37]. However, these aspects have not yet been specifically investigated in the current literature.

The expected general benefits of the navigated suture button procedure, such as being a really minimally invasive procedure with precise drill tunnel placement and the ability to check the AC joint position exactly, could thus be confirmed in this study.

Probably the most well-known disadvantage of all surgical procedures used to date to stabilize AC joint dislocations, such as the hook plate or the open and arthroscopically assisted suture button method, is recurrent instability with loss of reduction in the vertical and horizontal direction [11, 30, 31, 38, 39]. This study also showed a loss of AC joint reduction in the vertical direction from clavicle to coracoid process from a mean of 38.8 mm to 41.1 mm within the first 3 months after surgery (an additive horizontal AC cerclage or two suture button systems in the V-technique were not used in this study, but a slight AC joint overcorrection was aimed for when tightening the implants). Although not significant, the 2.3 mm of elongation had occurred without detectable malalignment, implant failure or fracture. This phenomenon, which is mostly radiologically conspicuous but sometimes also clinically correlatable, is usually asymptomatic without significant pain or functional impairment [40]. Surgical revision has not yet been necessary in this study due to the obviously small number of patients and relatively short follow-up period.

An existing barrier to the general use of the new method is likely to be the availability of the system requirements in the medical care institutions. Probably not every hospital is yet equipped with a carbon table, a mobile C-arm image intensifier with 3D capability and image-based computer navigation. Another disadvantage of this method is the higher radiation exposure for patients due to the 3D DVT scan, which is why the best possible shielding of sensitive tissue and organs must be ensured.

Limitations of this study are the small number of patients and the short follow-up period.

To summarise image-guided 3D C-arm navigated AC joint suture button stabilisation is feasible in everyday surgical practice. Nevertheless further prospective studies with a larger number of patients and long-term follow-up as well as a direct comparison of navigation with the established methods of the arthroscopic suture button procedure and the hook plate to investigate complaints, function and stability are necessary. It may also be useful to expand the navigated technique by adding an additional AC horizontal cerclage or a second CC suture button system to increase stability.

## Supplementary Information

Below is the link to the electronic supplementary material.

Supplementary file1 (PNG 364 KB)

## Data Availability

No datasets were generated or analysed during the current study.
