# Supplementary figures and images for: 3D C-arm navigated suture button implantation for AC joint dislocations - the pilot study
Source: Eur J Trauma Emerg Surg. 2024 Jul 12;50(5):2431–9. doi: 10.1007/s00068-024-02582-z (PMC11599409; doi:10.1007/s00068-024-02582-z)

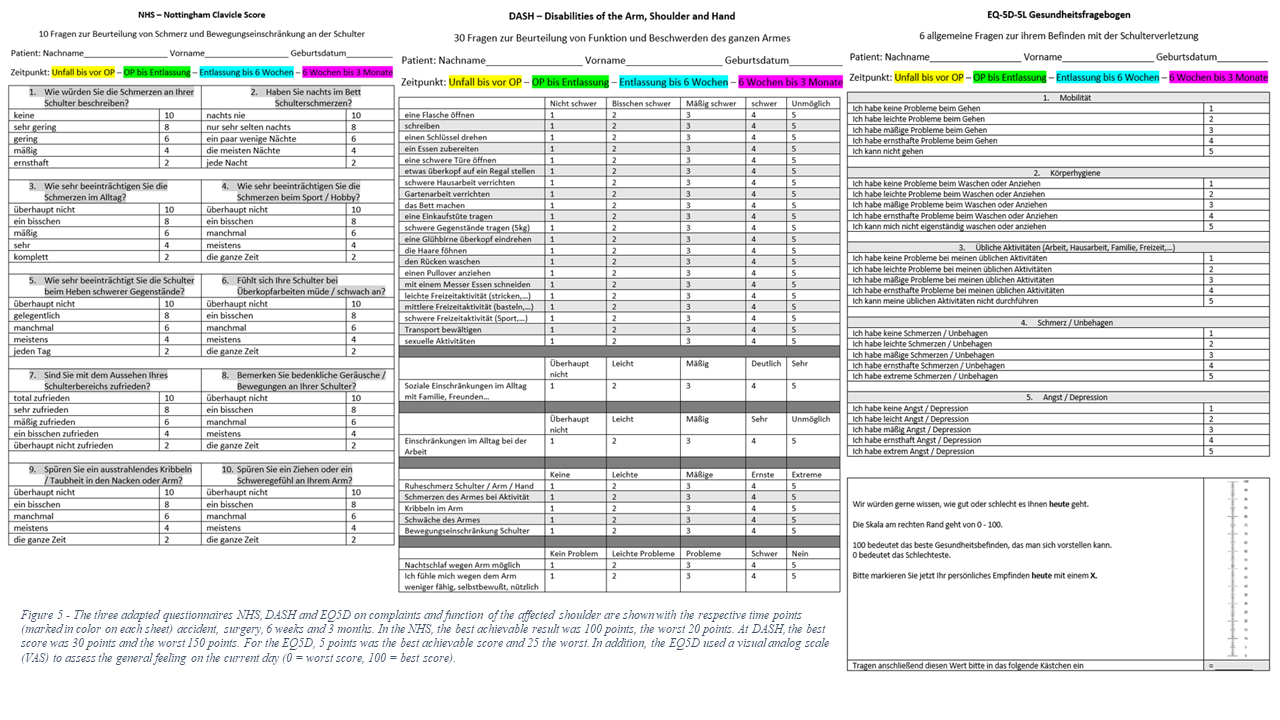

Supplement: Supplementary file 1 — Supplementary file1 (PNG 364 KB) [file 68_2024_2582_MOESM1_ESM.png]
